# Case Report: Histochemical and immunohistochemical characterization of a canine giant cell bone tumor in lumbar spine

**DOI:** 10.3389/fvets.2026.1756975

**Published:** 2026-03-12

**Authors:** María Victoria Soto-López, Miguel Fernández, Miguel Fuertes, José Espinosa, Lorena Millán-Varela, Iván Prada, María Carmen Ferreras

**Affiliations:** 1 Hospital Veterinario, Departamento de Medicina, Cirugía y Anatomía Veterinaria, Facultad de Veterinaria, Universidad de León, León, Spain, León, Spain; 2 Departamento de Sanidad Animal, Facultad de Veterinaria, Campus de Vegazana, Universidad de León, León, Spain; 3 Instituto de Ganadería de Montaña (CSIC-Universidad de León), León, Spain

**Keywords:** clinical case, dog, giant cell tumour, lumbar spine, pathology, oncology, internal medicine

## Abstract

A 10-year-old sexually intact male Poodle dog, weighing 14 kg, was referred with abnormal gait (ataxia) and antalgic posture. The dog had a medical history of previous trauma and prostatitis diagnosed six months ago. Serum biochemical analysis showed elevated alkaline phosphatase and alanine aminotransferase levels. Neurological examination revealed hind limb dragging, body tilt, delayed proprioception—particularly affecting the left hind limb—and pain upon palpation of the lumbar region. Imaging diagnostic made evident a mass and a significant osteolysis of the second lumbar vertebra (L2), particularly on the left side of the vertebral body, extending into the first (L1) and third (L3) lumbar vertebrae. Necropsy findings confirmed the presence of a nodular soft mass below the left kidney and a firm mass compressing the medullary canal at L1-L3. Both masses exhibited whitish areas interspersed with blood-filled spaces. The tumor comprised numerous multinucleated giant cells of the osteoclastic type (positive for tartrate-resistant acid phosphatase- TRAP- and lysozyme), evenly distributed among mononuclear cells (both rounded and spindle-shaped). Alpha smooth muscle actin (SMA) was expressed in mononuclear cells, while IBA-1 staining highlighted mononuclear histiocytic cells. The final diagnosis was a primary lumbar extradural giant cell tumor of bone.

## 1 Introduction

Giant cell tumour of the bone (GCTB) is a mesenchymal neoplasm that, according to the WHO classification, usually presents with locally aggressive features, behaving as a mass that progressively enlarges and destroys the bone, invading surrounding structures and rarely metastasizing (1). In veterinary medicine, GCTB is considered a rare neoplasm and has been sporadically reported in different animal species (2–6). In dogs, it has been described mainly in appendicular bones, including the metacarpal bone, humerus, tibia, accessory carpal bone, scapula and zygomatic arch, whereas axial and spinal localizations are exceptional. GCBT has been reported in the rib, femur, tibia and lumbar vertebra in cats and isolated cases have also been described in birds (7). Although metastases have been described in GCTB, lung metastases are considered an uncommon observation. In human medicine, GCTB accounts for 5% of all primary bone tumours in the mature skeleton (8, 9). However, in dogs, comprehensive descriptions combining clinical, imaging, pathological and immunohistochemical features of spinal GCTB are lacking.

## 2 Case description

A 10-year-old, sexually intact male Poodle dog, weighing 14 kg, was referred to the Veterinary Hospital of the University of León, Spain, with abnormal gait (ataxia) and antalgic posture. Other medical history included prostatitis, which was well controlled with osaterone acetate and firocoxib. The blood cell count was within reference limits. In the serum biochemical profile, alkaline phosphatase (ALP) (471 U/L, reference range, 20 to 150 U/L) and alanine aminotransferase (ALT) (624 U/L, reference range, 10 to 118 U/L) concentrations were increased.

On the neurological examination, the dog dragged the hind limbs, showed body tilt, and delayed proprioception, that was worse especially in the left hind limb, and showed pain on palpation along the lumbar region.

Radiographs showed marked osteolysis of lumbar vertebra 2 (L2), especially on the left side of the vertebral body, extending to L1 and L3 (Supplementary Figure 1). Magnetic resonance imaging (MRI) detected a mass with a multicystic appearance growing ventrally, towards the neighboring soft tissues (kidney and left adrenal), and dorsal, invading the longissimus lumbar muscle (Supplementary Figure 2). Given the extent of the lesion, the severity of neurological deficits and the poor prognosis, no therapeutic intervention was attempted. The dog was humanely euthanized shortly after imaging diagnosis and severe deterioration of health status, therefore there was no period of clinical follow-up. The animal was humanely euthanized and submitted for necropsy.

### 2.1 Gross findings

**Figure 1:**
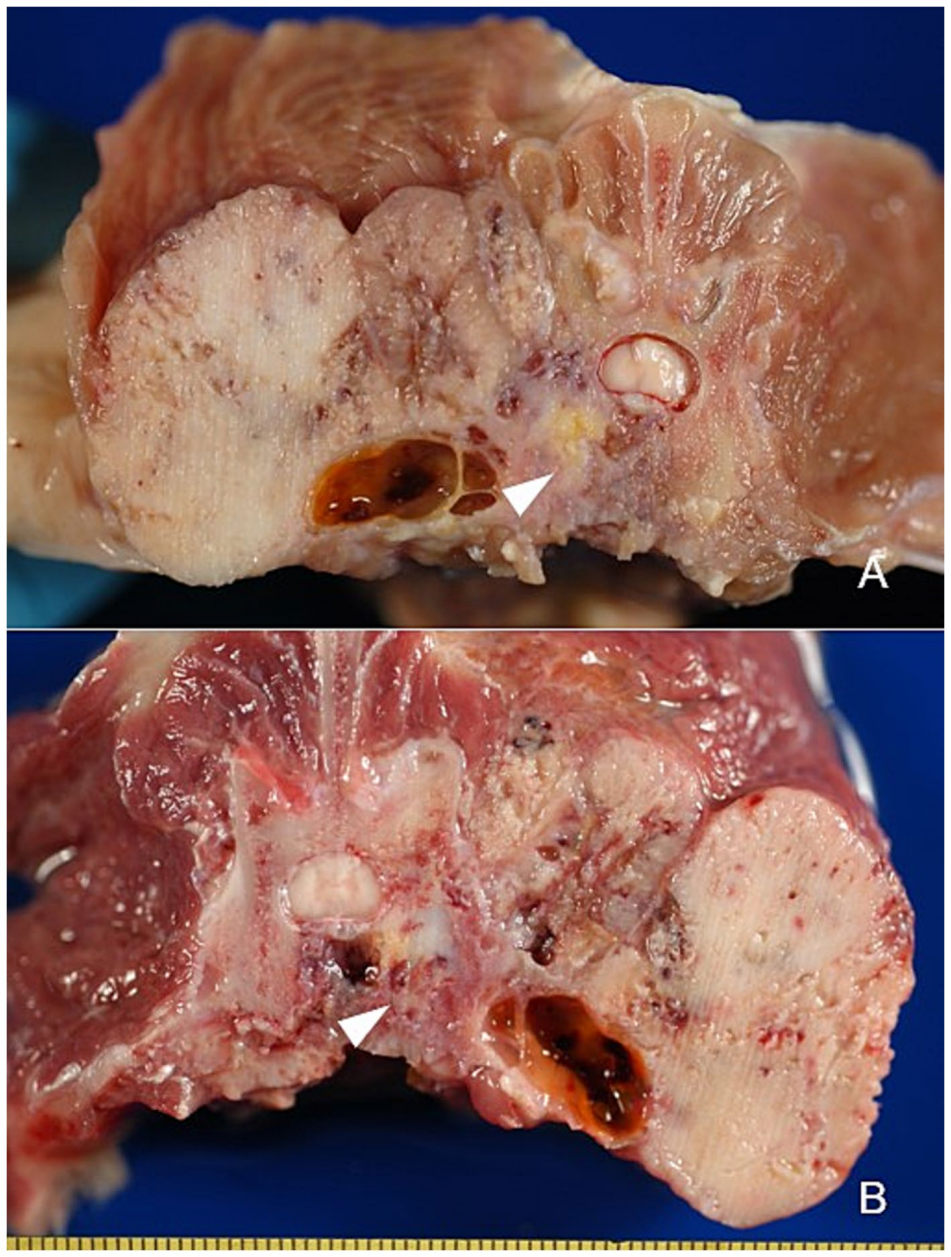
Eccentric osteolysis of L2 **(A)** and L3 **(B)** (arrowheads).

At necropsy, the existence of a nodular soft mass of approximately 8x6x4 cm located below the left kidney and another firm in the vicinity of 6x4x4 cm that compressed the medullary canal at L1-L3 and infiltrated dorsally between paravertebral muscle tissue, was confirmed (Supplementary Figure 3A). Both masses showed whitish areas alternated with blood-filled spaces (Supplementary Figure 3B). The transverse sectioning of the body of L2 exposed a white-coloured mass with cystic bloody space that compressed the spinal cord (Figure 1A). Transverse section of body of L3 revealed a similar area with cystic spaces without compression of the spinal cord (Figure 1B). No significant pathological findings related to the tumour were observed in the remaining sampled organs. Organ sampling was performed systematically during necropsy to evaluate the presence of metastatic disease, with particular attention to organs commonly affected by metastases, including lung, liver, spleen and brain.

### 2.2 Microscopic findings

Tissue samples from the body of L2, L3, and both tissue masses as well as other internal organs (liver, lung, heart, intestine, stomach, prostate, adrenal gland, kidney, spleen, pancreas, brain) were routinely fixed in 10% buffered formalin and embedded in paraffin wax. The vertebral tissue was previously decalcified according to the formic acid sodium citrate method. Paraffin-embedded tissue samples were stained with hematoxylin and eosin (H-E) and Masson-Goldner trichrome stains for histologic examination. The mitotic index was estimated by counting the number of total mitoses per mm^2^. Additionally, tumour samples were examined histochemically for the osteoclast-specific enzyme tartrate resistant acid phosphatase (TRAP) using an acid phosphatase, leucocyte kit (Sigma-Aldrich, St Louis, MO, USA).

Histologically, the tumour masses were partially surrounded by a thick connective tissue capsule and were highly vascularized, with areas of hemorrhage and even thrombus formation, causing multifocal bone lysis and invasion of the bone marrow.

**Figure 2:**
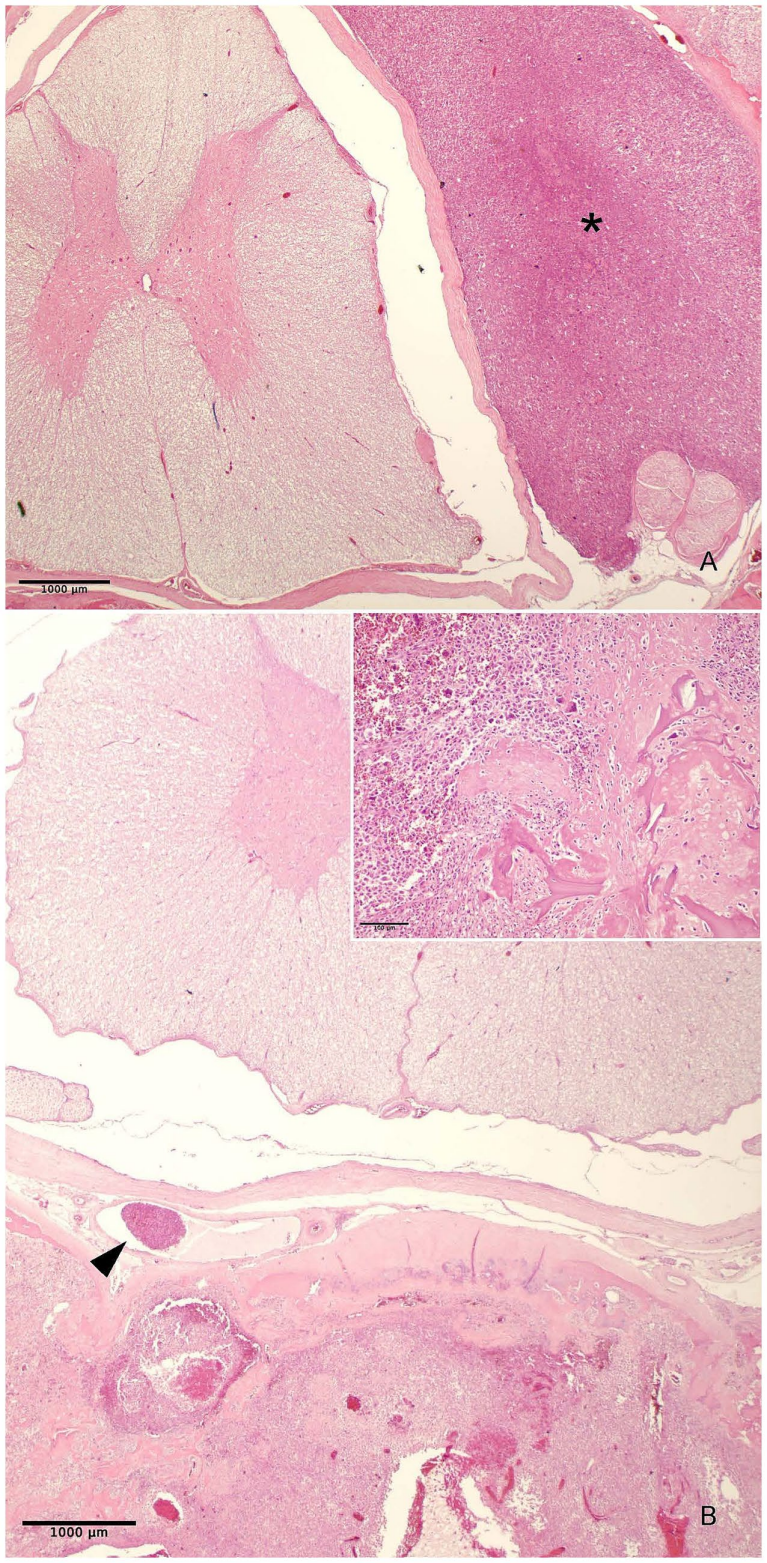
Photomicrographs of sections of the L2 and L3, H&E **(A,B)**. Invasion of the tumour mass into the epidural space with compression of the spinal cord and spinal nerves; bar = 1,000 μm (asterisk) **(A)**. Eccentric osteolysis of L3 without spinal cord compression (inset; bar = 100 μm), with tumour invasion of the spinal canal vessels (arrowhead) and the vertebral body; bar = 1,000 μm **(B)**.

**Figure 3:**
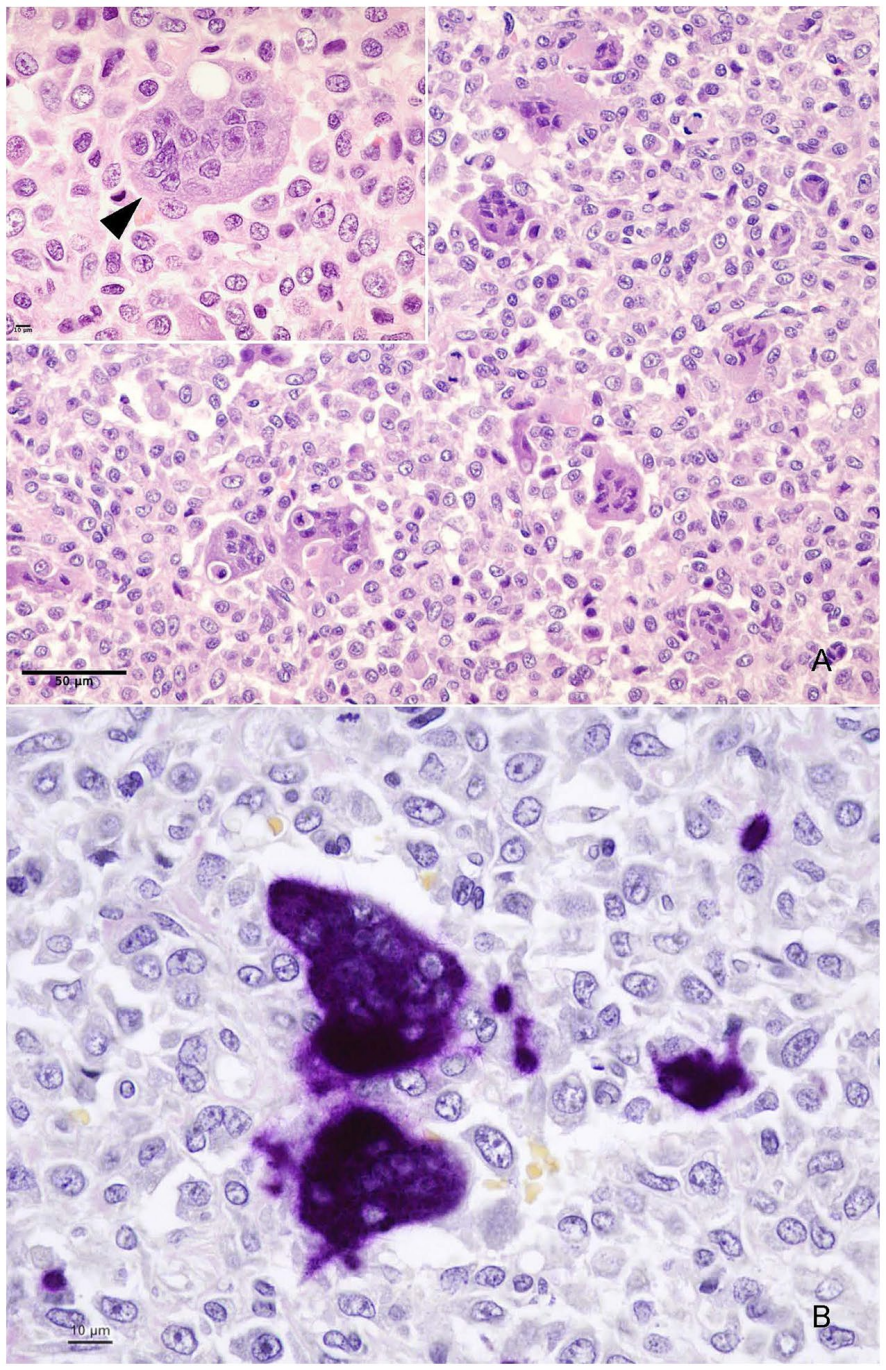
The correct caption is: Multinucleated giant cells (A-inset; bar = 10 μm) dispersed among mononucleated cells (fusiform and ovoid) with minimal nuclear atypia, H&E; bar = 50 μm **(A)**. Multinucleated, osteoclast-like giant cells stain intensely with tartrate-resistant acid phosphatase (TRAP), an osteoclastic phenotype marker; bar = 10 μm **(B)**.

At the L2 level the tumour tissue infiltrated the spinal canal and pressed onto the spinal cord and spinal nerves (Figure 2A). It also invaded the periosteum and extended to the surrounding striated muscle. At the L3 level, without spinal cord compression, the tumour cells invaded the spinal canal vessels and the vertebral body (Figure 2B). Marked bone necrosis and tumour invasion of the bone marrow were observed (Supplementary Figures 4, 5). The tumour consisted of numerous multinucleated giant cells (up to 33 nuclei) of the osteoclastic type (positive for tartrate-resistant acid phosphatase -TRAP-), evenly distributed among mononucleated cells (rounded or spindle-shaped) with mid cellular atypia (Figure 3A, inset; Figure 3B). These cells were seen within vessels in the epidural venous plexus or in relation to hemorrhagic areas (Figure 2B). The mitotic count was scarce, ranging from 0 to 2.5 mitosis per mm^2^ (median 1.9 mitoses per mm^2^) and limited to mononuclear cells. Foci of osteoid and areas of coagulation necrosis are also seen.

No metastatic lesions were observed.

### 2.3 Immunohistochemistry

Immunohistochemical labelling of formalin-fixed paraffin-embedded 3.5-μm-thick serial tissue sections was performed to determine the phenotype of the cells. The antibody panel included vimentin V9 (M0725; Dako), alpha smooth muscle actin (SMA) 1A4 (M0851; Dako), lysozyme EC 3.2.1.17 (A0099; Dako) and ionised calcium-binding adaptor molecule-1 (IBA-1) (019e19741, Wako). Epitope retrievals were accomplished in Tris-based solution (PT-Link System, Agilent Technologies) at pH = 9 for alpha SMA and pH = 6 for vimentin, lysozyme and IBA-1. In addition, slides were immersed in a 3% H_2_O_2_ in methanol solution for 30 min in the dark at room temperature and incubated overnight at 4 °C with primary antibodies diluted (vimentin 1:1000; alpha SMA 1:100; lysozyme 1:250; Iba-1 1:2000) in a phosphate-buffered saline in a humidified chamber. The next day, after washing, incubation with a secondary antibody for 40 min and with 3,3-diaminobenzidine (DAB; Agilent Technologies) for 5 min was performed. Finally, slides were rinsed in tap water and counterstained with Mayer’s haematoxylin for 10 s. Negative and positive controls, for the primary antibodies, were included. Positive control tissues included canine lymph node for IBA-1 and lysozyme, and canine smooth muscle tissue for *α*-SMA, while negative controls were obtained by omission of the primary antibody. The results of IHC study in each of the neoplastic cell subtypes are summarised in Table 1.

**Table 1:** Expression of the markers used in the different cell types according to the percentage of staining observed.

Antibody marker	Clone/brand	D	Cell type		
			Mononuclear stromal (spindle cells)	Monocytes/Macrophages (histiocytic cells)	Osteoclasts-like (giant cells)
Vimentin V9	M0725/Dako	1:1000	3	3	3
Iba-1	019e19741/Wako	1:2000	0	2	0
Lysozyme EC 3.2.1.17	A0099/Dako	1:250	0	2	2
α-SMA 1A4	M0851/Dako	1:100	1	1	0
H3.3 G34W	RM263; RevMAb	1:1500	0	0	0
TRAP (h)	–		0	1	4

0 = none, 1 = less than 25%, 2 = between 25–50%, 3 = 50–75%, 4 = more than 75%. h = histochemistry; D = Dilution.

**Figure 4:**
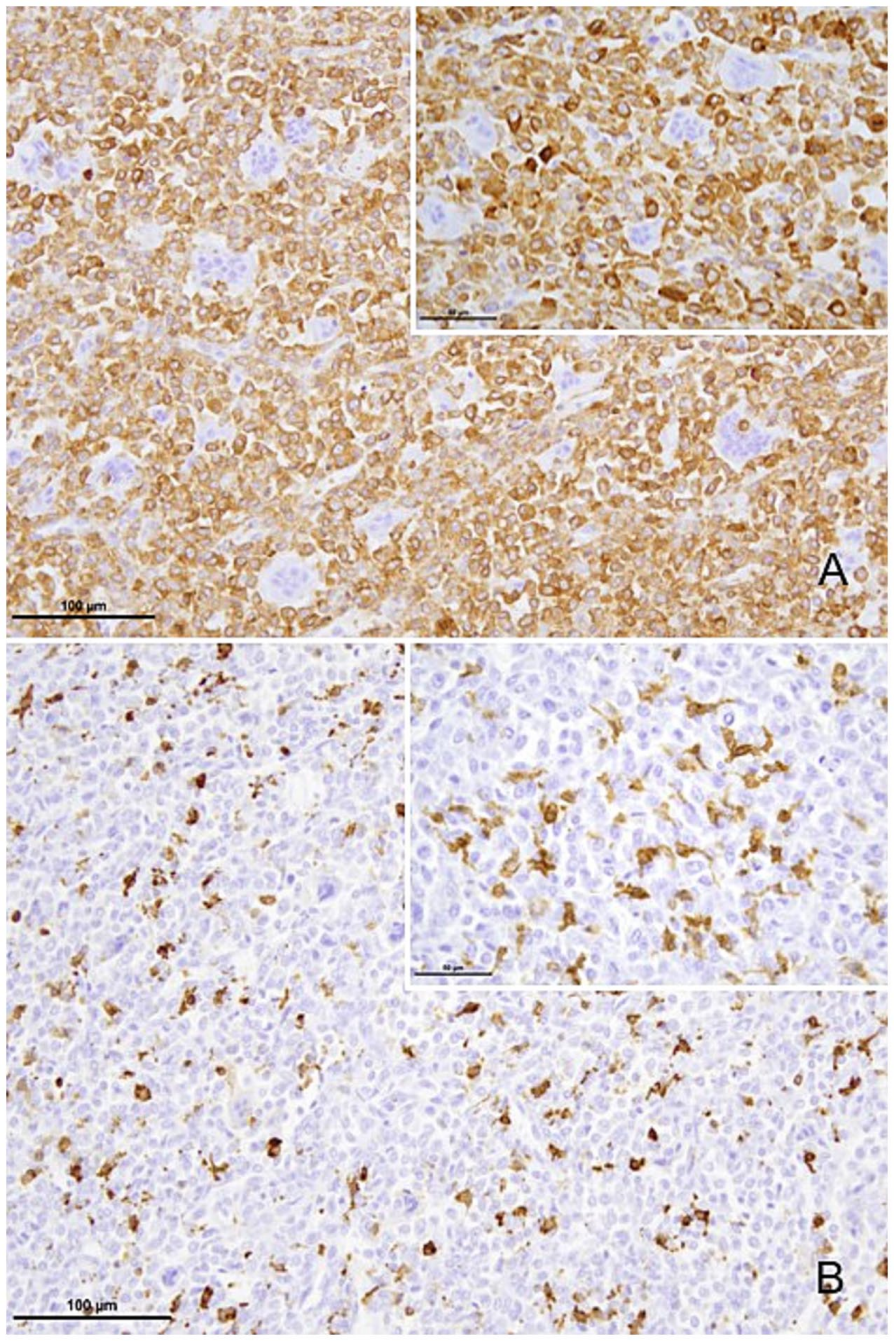
Anti alpha SMA antibody-stained mononuclear cells in the tumor; bar = 100 μm **(A)**, bar = 50 μm (**A** inset). Positive immunollabeling for anti-Iba 1 antibody in mononuclear histiocytic cells including their ramifications. Mononuclear tumor cells in mitosis are unstained (arrowhead); bar = 100 μm **(B)**, bar = 50 μm (**B** inset).

The three cell types of the tumour: spindle cells like mononuclear stromal cells, cells resembling the monocyte/macrophages and cells with morphologies like those of osteoclasts were positive for vimentin and only the last two cell types expressed lysozyme. Alpha SMA was expressed in the cytoplasm of about 90% of mononuclear cells while the giant cells were negative (Figure 4A, inset). The IBA-1 antibody was clearly expressed in the membrane and cytoplasm of rounded mononuclear cells in the tumour (Figure 4B, inset). These IBA-1 positive cells were more abundant in areas of tumour proliferation (Table 1). Lysozyme expression was irregular, being scarce positivity in mononuclear and some osteoclast giant cells (Supplementary Figure 6). Some formalin-fixed paraffin-embedded tissue sections of the present case were kindly stained in the Department of Histopathology of the Royal National Orthopaedic Hospital, Stanmore, UK against the anti-histone H3.3 G34W antibody, which is validated to be used in human tissue. However, immunohistochemistry for the H3.3 G34W mutation, performed on selected sections, showed no nuclear immunoreactivity in this tumour cells.

## 3 Discussion

Giant cell tumour of the bone (GCTB) is a locally aggressive mesenchymal neoplasm characterized by progressive osteolytic growth, invasion of adjacent structures and a low metastatic potential (10, 11). In the present report, a primary lumbar extradural GCTB involving three contiguous vertebrae (L1–L3) was diagnosed in a dog based on clinical presentation, imaging findings, gross pathology, histological examination, histochemical staining and immunohistochemical characterization.

In dogs, GCTB is considered a rare tumour and most reported cases involve appendicular bones such as the metacarpal bone (12), humerus (13), zygomatic arch (14), scapula (6), accessory carpal bone (15) and tibia (3) with occasional metastases in the lung (10). GCTB occurs less frequently in the axial skeleton (11) and few reported cases involving the occipital condyle in the skull (16).

For the moment, no previous canine reports have provided a detailed pathological and immunohistochemical characterization of a spinal GCTB. In the present case, the tumour exhibited the classical histological features of GCTB, including numerous multinucleated osteoclast-like giant cells evenly distributed among mononuclear stromal and histiocytic cells, associated with extensive bone destruction and invasion of adjacent soft tissues.

Immunohistochemical and histochemical analyses allowed a clear distinction between the different cellular populations within the tumour. In the present work, the mononuclear tumour cells stained positive for antibodies against vimentin and *α*-SMA, whereas the last antibody exhibited a negative reaction on the osteoclast-like giant cells. Expression of α-SMA is confined to blood vessels in normal adult cortical and cancellous bone, as previously reported (17). However, this antibody is differentially expressed by mononuclear stromal cells in primary tumours and tumour-like lesions of bones, suggesting that identification of these cells may be useful in the differential diagnosis of primary bone tumours (17). In the present cases and previous reported cases, this antibody was also noted in mononuclear but not in multinucleated giant cells, with moderate/strong reaction, in primary, recurrent and pulmonary metastatic GCTB without specific differences (17–19). Non-neoplastic mononuclear histiocytic cells are considered to belong to the monocyte–macrophage lineage, either reactive macrophages or osteoclast precursors (20). Mononuclear histiocytic cells showed immunoreactivity for vimentin, lysozyme and IBA-1, a general macrophage/microglial marker expressed in the membrane and cytoplasm of activated monocyte–macrophage lineage cells (21). In contrast, neoplastic mononuclear cells and osteoclast-like giant cells were IBA-1 negative. As these cell populations are indistinguishable on hematoxylin–eosin-stained sections (22), the intense IBA-1 immunopositivity observed in this study proved useful for their accurate identification in GCTB. Reactive osteoclast-like giant cells showed a strong enzyme histochemical reaction for tartrate-resistant acid phosphatase (TRAP) in the present case. Some mononuclear cells, which also stained TRAP positively, could be the precursors of the giant cells, as has been suggested (20). The biological behaviour of GCTB is largely driven by osteoclastogenesis, with osteoclast-like giant cells expressing bone-resorbing enzymes such as TRAP (23). Fusion of neoplastic mononuclear cells leads to the formation of multinucleated giant cells responsible for osteolysis, which underlies the large tumour size and local aggressiveness of GCTB (20, 24, 25). These features support the diagnostic value of histochemical and immunohistochemical characterization in canine GCTB. In veterinary medicine, GCTB has also been sporadically reported in cats, affecting bones such as the rib (26), tibia (5), femur (2) and, more rarely, the lumbar vertebra (4). In birds, one case in the scapula was documented (7). These cases shared several histological and biological features with those observed in dogs, including marked osteolysis, locally aggressive growth and abundant osteoclast-like giant cells, although metastatic behaviour. Spinal involvement remains exceptional in companion animals, emphasizing the unusual anatomical location and extent of the tumour described in the present canine case.

In human medicine, GCTB most commonly affects the epiphyseal region of long bones, whereas involvement of the axial skeleton is uncommon (8–10). Spinal localization is rare and typically presents as an eccentric, lytic lesion involving one or more contiguous vertebrae, with extension into the spinal canal and paravertebral soft tissues, often resulting in pain and neurological deficits.

The eccentric, osteolytic lesion expanded above the vertebral body to the spinal canal and paravertebral soft tissues, was similar to previous descriptions in humans (8, 9, 27, 28). The tumour produces localised pain and neurological signs due to root and spinal injury (27, 28).

Histologically, GCTB contains an admixture of ovoid to spindly mononuclear tumour cells, non-neoplastic mononuclear histiocytic cells, and numerous reactive osteoclast-like giant cells. Mononuclear cells with an osteoblastic precursor phenotype are the true neoplastic element of GCTB, whereas mononuclear histiocytic cells and osteoclast-like multinucleated giant cells are considered non-neoplastic elements (22). It is a hypervascular tumour that, despite its local aggressiveness, shows slight cellular atypia and, although it does invade vessels, as in the present case, its metastatic potential is very weak (18, 29).

The spinal location is exceptional (30) and in this case, the condition is usually monovertebral or plurivertebral, frequently contiguous, lytic, and eccentric (31). Only 2 to 5% of the GCTB cases have been found in the vertebra above the sacrum (32) and account approximately 0.1 to 0.25% of the bone tumours (33, 34). Several cases of GCTB of the third (35–37), fourth (38) and fifth lumbar vertebra (11, 29, 31) have been documented in the literature. Collapse of L3 vertebra with contiguous involvement of L2 and L4 with a large soft tissue component has been considered an unusual finding (28). In these cases, the tumour causes pain and osteolysis of the vertebral body with extension to the spinal canal (29, 36–38) and paravertebral soft tissues (11, 29, 38). Surgical treatment was carried out, and biopsy samples confirmed the diagnosis. Only in two cases (36, 38) was local tumour recurrence observed, which seems to be related to a higher risk of lung metastases (39).

From a biological perspective, GCTB is considered a predominantly osteoclastogenic stromal tumour, characterized by expansile osteolytic lesions and a high prevalence of multinucleated giant cells, which may account for up to 25% of the tumour cell population (13). Although osteoid production may occur in both GCTB and telangiectatic osteosarcoma, it is more prominent in osteosarcoma, which also shows greater cellular atypia (4, 40). In GCTB, mononuclear stromal cells represent the true neoplastic component and drive osteoclastogenesis through the recruitment and activation of osteolysis, leading to extensive bone resorption; the abundance of TRAP-positive giant cells and marked osteolysis observed in this case are consistent with this mechanism (4, 13, 40).

In human pathology, most GCTBs harbour mutations in the H3F3A gene encoding histone H3.3, and immunohistochemical detection of the H3.3 G34W mutant protein is widely used as a diagnostic tool (8, 10, 25). In the present canine case, H3.3 G34W immunoreactivity was absent, which may reflect species-related differences in histone H3.3 sequence or antibody cross-reactivity, or alternative pathogenetic mechanisms in canine GCTB, as molecular heterogeneity has been described even in humans (10, 25, 40, 41). Therefore, the lack of H3.3 G34W expression does not preclude the diagnosis, which was supported by the integrated clinical, morphological, histochemical and immunohistochemical findings. A limitation of the present case report is that the definitive diagnosis was achieved only after post-mortem examination. Therefore, the potential *in vivo* diagnostic value of minimally invasive techniques, such as cytology, could not be assessed. Cytological samples could have been obtained after euthanasia, and their absence represents a limitation of this report.

## 4 Conclusion

This is the first detailed description of a lumbar spinal giant cell tumour of bone in a dog including an extended immunohistochemical characterization. This work describes a GCTB involving three contiguous lumbar vertebrae with a large soft tissue component, an unusual finding in veterinary medicine. The eccentric, osteolytic lesion expanded above the vertebral body to spinal canal and paravertebral soft tissues was similar to previous descriptions in humans.

## Data Availability

The raw data supporting the conclusions of this article will be made available by the authors, without undue reservation.

## Supplementary material

SUPPLEMENTARY FIGURE 1 Antero-posterior radiograph with eccentric osteolytic image mainly of the body of L2 (arrowhead).

SUPPLEMENTARY FIGURE 2 MRI: masses expanding into soft tissues in the lumbar area (arrowhead).

SUPPLEMENTARY FIGURE 3 Macroscopic image of the nodular soft masses (discontinuous white line) **(A)**. Cross section of the tumour, highly vascularized (arrowhead) **(B)**.

SUPPLEMENTARY FIGURE 4 Multinucleated giant cells in blood vessels and mitosis with some lysis of osteoclasts; bar = 50 μm.

SUPPLEMENTARY FIGURE 5 Osteonecrosis and tumour invasion of the bone marrow can be seen. Masson-Goldner trichrome stain, bar = 1000 μm **(A)**; bar = 50 μm **(B)**.

SUPPLEMENTARY FIGURE 6 Immunohistochemistry staining against lysozyme. Mononuclear cells are slightly stained; bar (inset) = 50 μm; bar = 100 μm.
