# Supplementary figures and images for: Case Report: Histochemical and immunohistochemical characterization of a canine giant cell bone tumor in lumbar spine
Source: Front Vet Sci. 2026 Mar 12;13:1756975. doi: 10.3389/fvets.2026.1756975 (PMC13019699; doi:10.3389/fvets.2026.1756975)

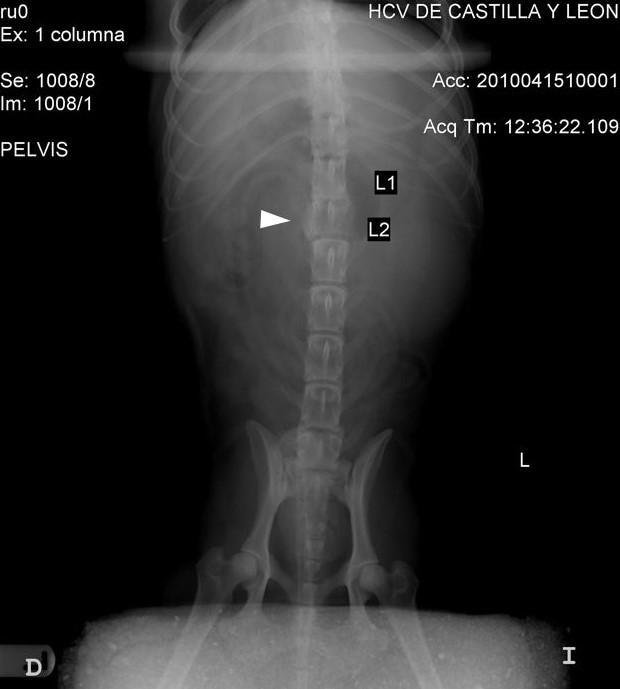

Supplement: SUPPLEMENTARY FIGURE 1 — Antero-posterior radiograph with eccentric osteolytic image mainly of the body of L2 (arrowhead). [file Image_1.jpeg]

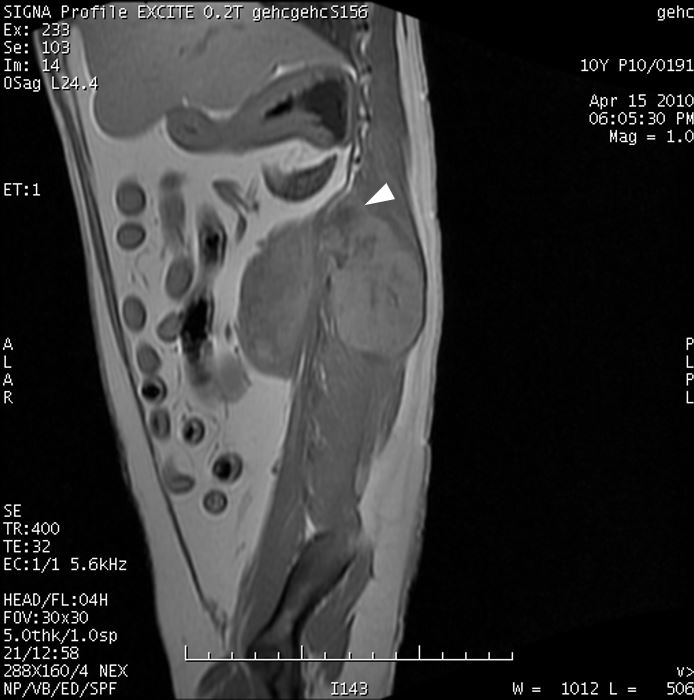

Supplement: SUPPLEMENTARY FIGURE 2 — MRI: masses expanding into soft tissues in the lumbar area (arrowhead). [file Image_2.jpeg]

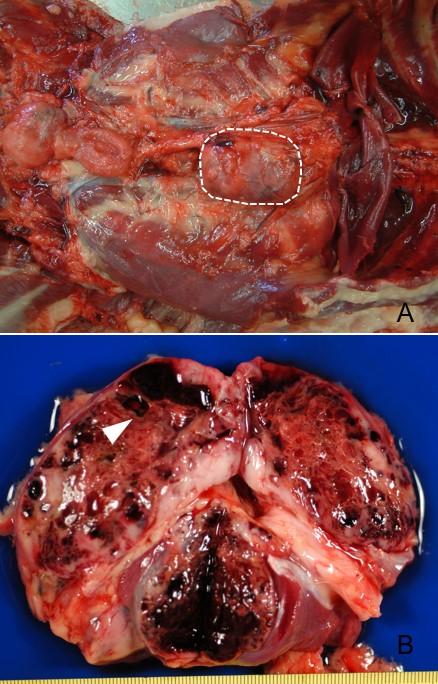

Supplement: SUPPLEMENTARY FIGURE 3 — Macroscopic image of the nodular soft masses (discontinuous white line) (A). Cross section of the tumour, highly vascularized (arrowhead) (B). [file Image_3.jpeg]

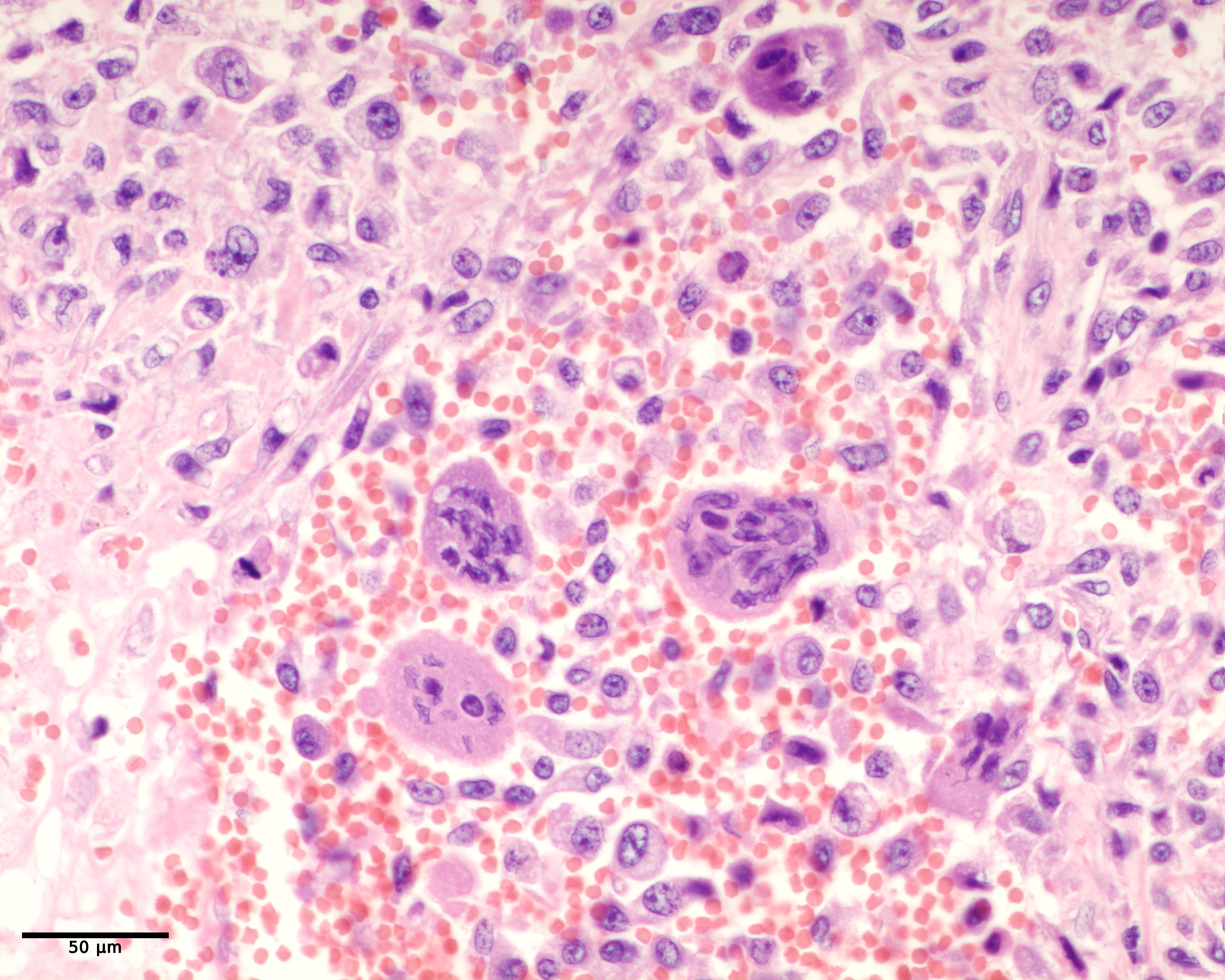

Supplement: SUPPLEMENTARY FIGURE 4 — Multinucleated giant cells in blood vessels and mitosis with some lysis of osteoclasts; bar = 50 μm. [file Image_4.jpeg]

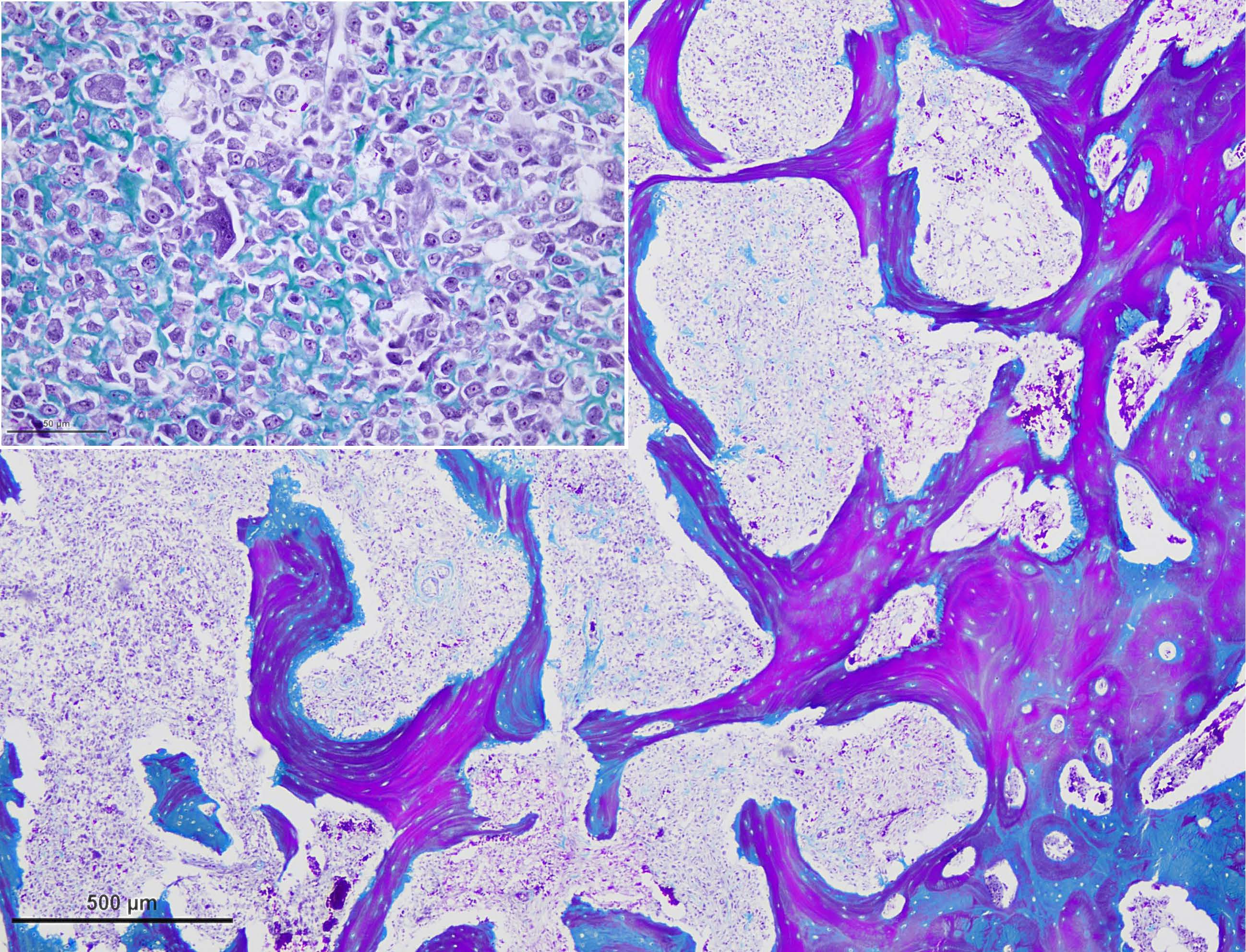

Supplement: SUPPLEMENTARY FIGURE 5 — Osteonecrosis and tumour invasion of the bone marrow can be seen. Masson-Goldner trichrome stain, bar = 1000 μm (A); bar = 50 μm (B). [file Image_5.jpeg]

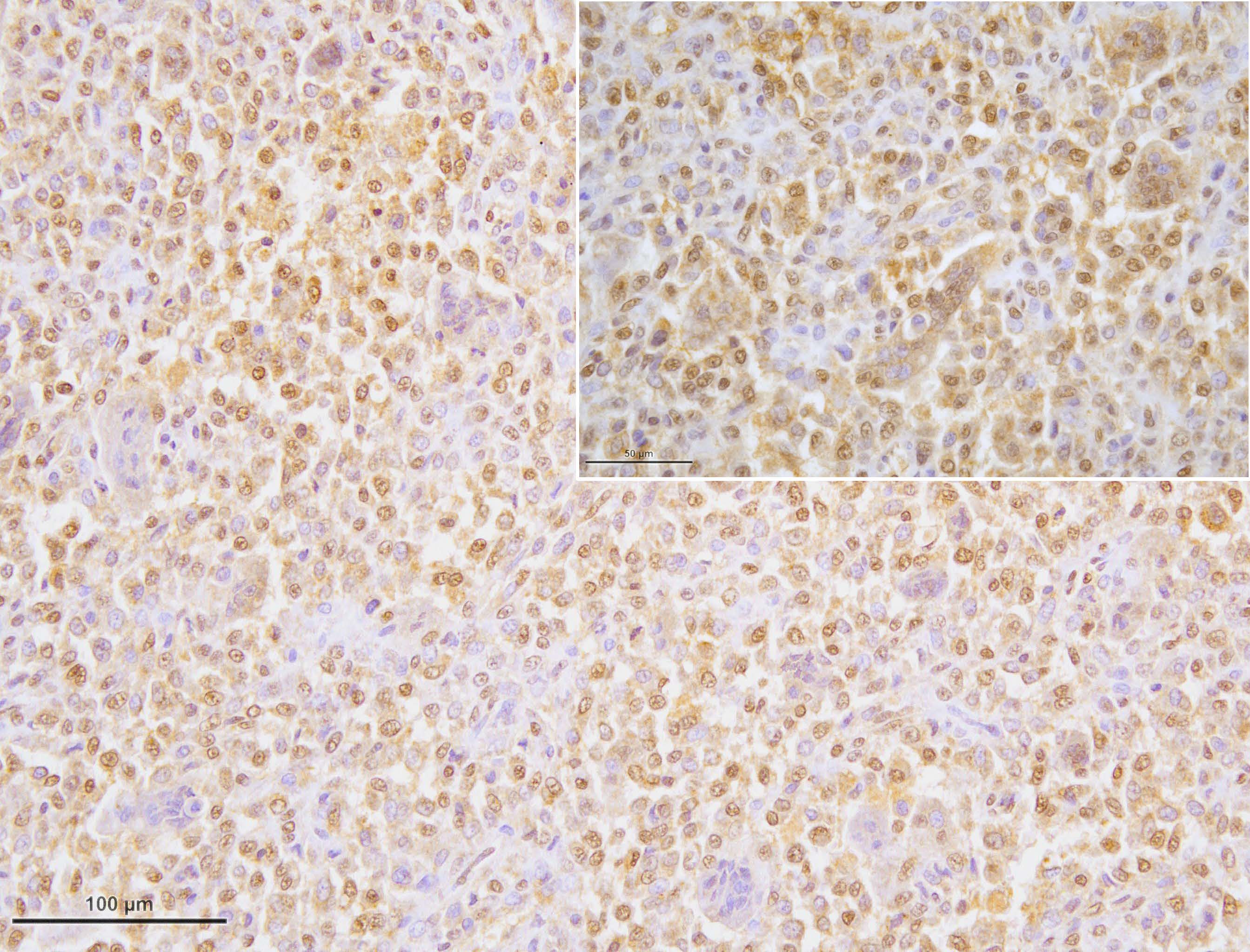

Supplement: SUPPLEMENTARY FIGURE 6 — Immunohistochemistry staining against lysozyme. Mononuclear cells are slightly stained; bar (inset) = 50 μm; bar = 100 μm. [file Image_6.jpeg]
